# Supplementary figures and images for: Evaluation and Comparison of Multi-Omics Data Integration Methods for Subtyping of Cutaneous Melanoma
Source: Biomedicines. 2022 Dec 13;10(12):3240. doi: 10.3390/biomedicines10123240 (PMC9775581; doi:10.3390/biomedicines10123240)

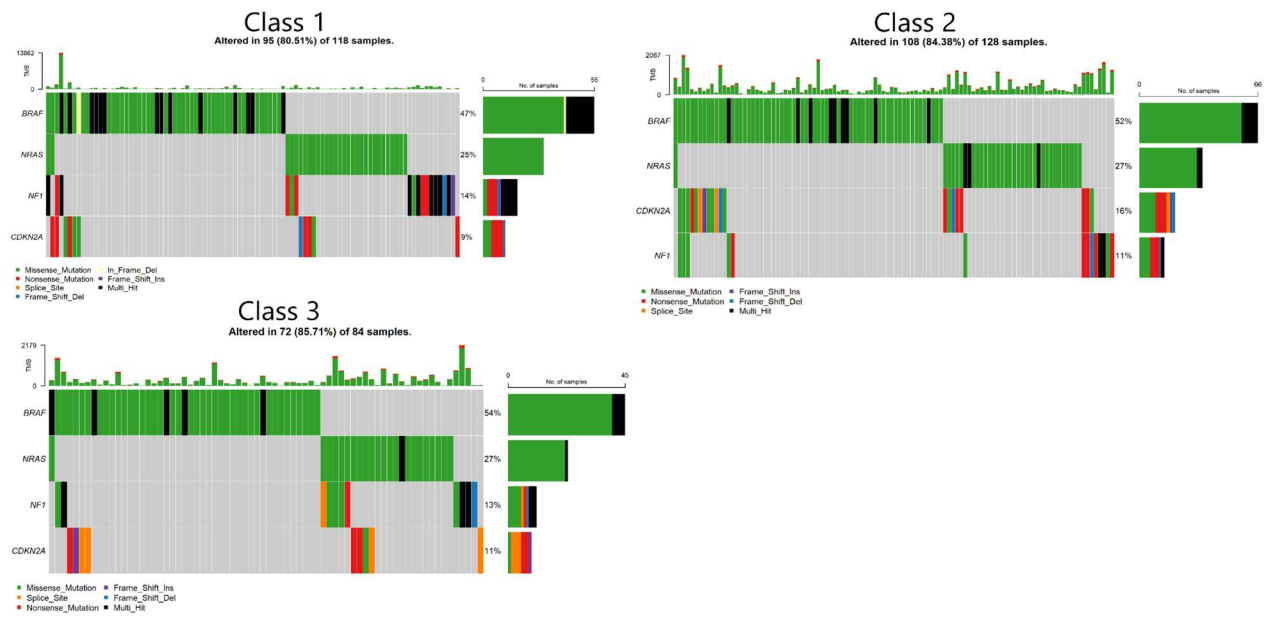

**Figure S1:** variant distribution in JSVD classes.

Supplement: Supplementary file 1 [file biomedicines-10-03240-s001.zip › biomedicines-2035955-supplementary-12.19/figure s1.pdf]
